# Unintentionally retained lap sponge mimicking an ovarian cyst two years after Caesarean section in a 37-year old patient: case report of a rare “never event” in Sudan

**DOI:** 10.1186/s13037-024-00407-x

**Published:** 2024-08-16

**Authors:** Hagir Osman Ahmed Elamin, M. Sayed Masoud, Khattab Saeed Elkhazin Mohamed Ali, Hiba Awadelkareem Osman Fadl, Abdelrahman Hamza Abdelmoneim Hamza, Hind Abashar Mohamed Basheer, Mohamed Alfaraja

**Affiliations:** 1 Faculty of Medicine and Health Sciences, University of Bakht Alruda, Ad Duwaym, Sudan; 2 Faculty of Medicine and Health Sciences, University of Shendi, Shendi, Sudan; 3 Department of Haematology, Faculty of Medical Laboratory Sciences, Al-Neelain University, Khartoum, Sudan; 4 Senior Medical Laboratory Specialist, Saudi Commission for Health Specialties (SCFHS), Makkah, Kingdom of Saudi Arabia; 5 Clinical Immunology Resident, Medical Specialization Board (SMSB), Khartoum, Sudan; 6 Faculty of Medicine, Al-Neelain University, Khartoum, Sudan; 7 Department of Obstetrics and Gynaecology, Faculty of Medicine and Health Sciences, University of Bakht Alruda, Ad Duwaym, Sudan

**Keywords:** Gossypiboma, Cesarean section, Intestinal obstruction, Sudan

## Abstract

**Introduction:**

This case report reports an unusual occurrence of gossypiboma, which refers to the accidental retention of surgical materials like sponges in the peritoneal cavity. The term is derived from “gossypium” (cotton) and “boma” (place of concealment). Its incidence varies with surgical type, posing diagnostic challenges due to nonspecific symptoms and equivocal imaging. Despite its rarity, gossypiboma poses significant risks, including intestinal obstruction and abscess formation.

**Case presentation:**

A 37-year-old woman with ten previous pregnancies and an emergent caesarean section presented with abdominal pain. Examination and ultrasound suggested an ovarian cyst. During surgery, a 10 × 10 cm gauze-filled mass adherent to the ovary and jejunum was found. Postoperatively, she recovered well with no complications. The patient was treated with intravenous fluids and antibiotics for five days post-surgery and recovered without any complications. She was discharged from the hospital five days after the procedure.

**Conclusion:**

To the best of our knowledge, this is the first reported case of gossypiboma in Sudan in 2024, highlighting diagnostic challenges and the need for preventive protocols. Root cause analysis of accidents, enhanced training, application of advanced technologies and a collaborative culture in the operating room can prevent the occurrence of such incidents. This case underscores the importance of meticulous surgical protocols and continuous improvement in safety measures to prevent retained surgical items, ensuring patient safety and optimal outcomes.

## Introduction

The terms gossypium (meaning cotton) and boma (meaning place of concealment) are the sources of what is known as gossypiboma [1]. The phrase describes the unintentional placement of a cotton compress, or surgical sponge in the peritoneal cavity during operation [2]. The intraperitoneal cavity is where gossypibomas are most frequently found [3]. There is a 0.001–0.1% chance of leaving a foreign body in the surgical field after a single procedure, and 90% of the objects that are left behind are soft foreign bodies like gauze or surgical sponges [4]. However, the real number of cases is still underestimated. Depending on the type of surgical intervention, the incidence of retained foreign bodies varies: 17.69% for caesarean sections, 16.33% for abdominal hysterectomy, and 13.54% for exploratory laparotomies in the acute abdomen [5]. Patients with a higher body mass index, those undergoing emergent surgery, and circumstances where the surgical approach changes suddenly are more likely to have retained surgical items [6]. The clinical manifestations are nonspecific, and the imaging results are frequently equivocal; therefore, diagnosis is typically challenging [7]. The condition can manifest as intestinal blockage, peritonitis, adhesions, fistulas, abscesses, erosion into the gastrointestinal tract, or even passing through the rectum. Early identification of such a condition will guarantee prompt initiation of suitable care, lowering the morbidity and fatalities of these patients.

Ensuring the safety of patients in the operating room is complicated task as elucidated by previous review studies that assessed several cases of unintentionally retained surgical sponges (RSS) in a duration of several years, and founded twelve possible risk factors which could lead to this event. This multiple interrelated factors could explain why this event still recurring despite the effort put against it [8]. 

In our case, a gossypiboma that mimicked an ovarian cyst was diagnosed two years after a caesarean section.

## Case presentation

**Fig. 1:**
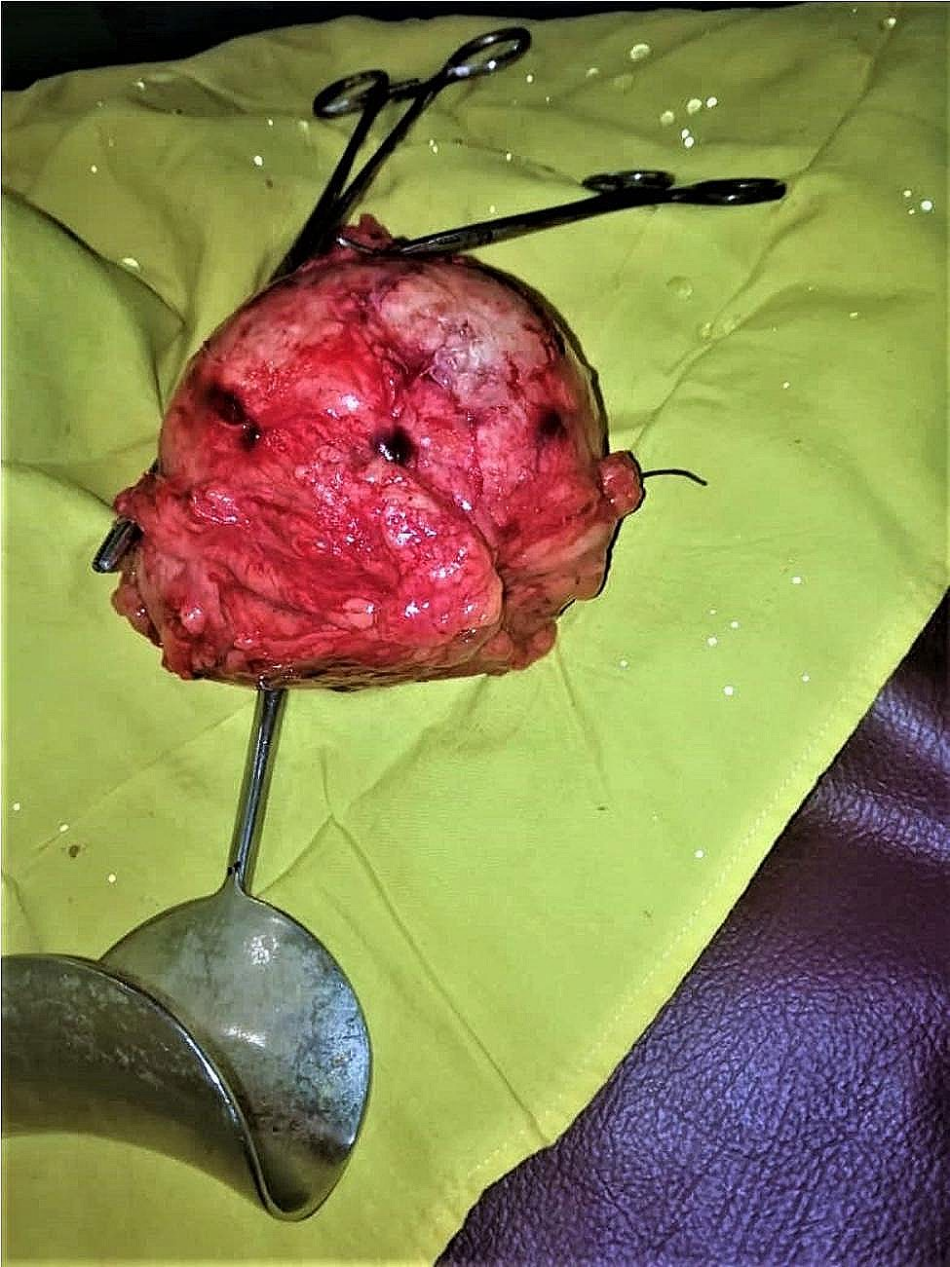
postoperative view of gossypiboma

**Fig. 2:**
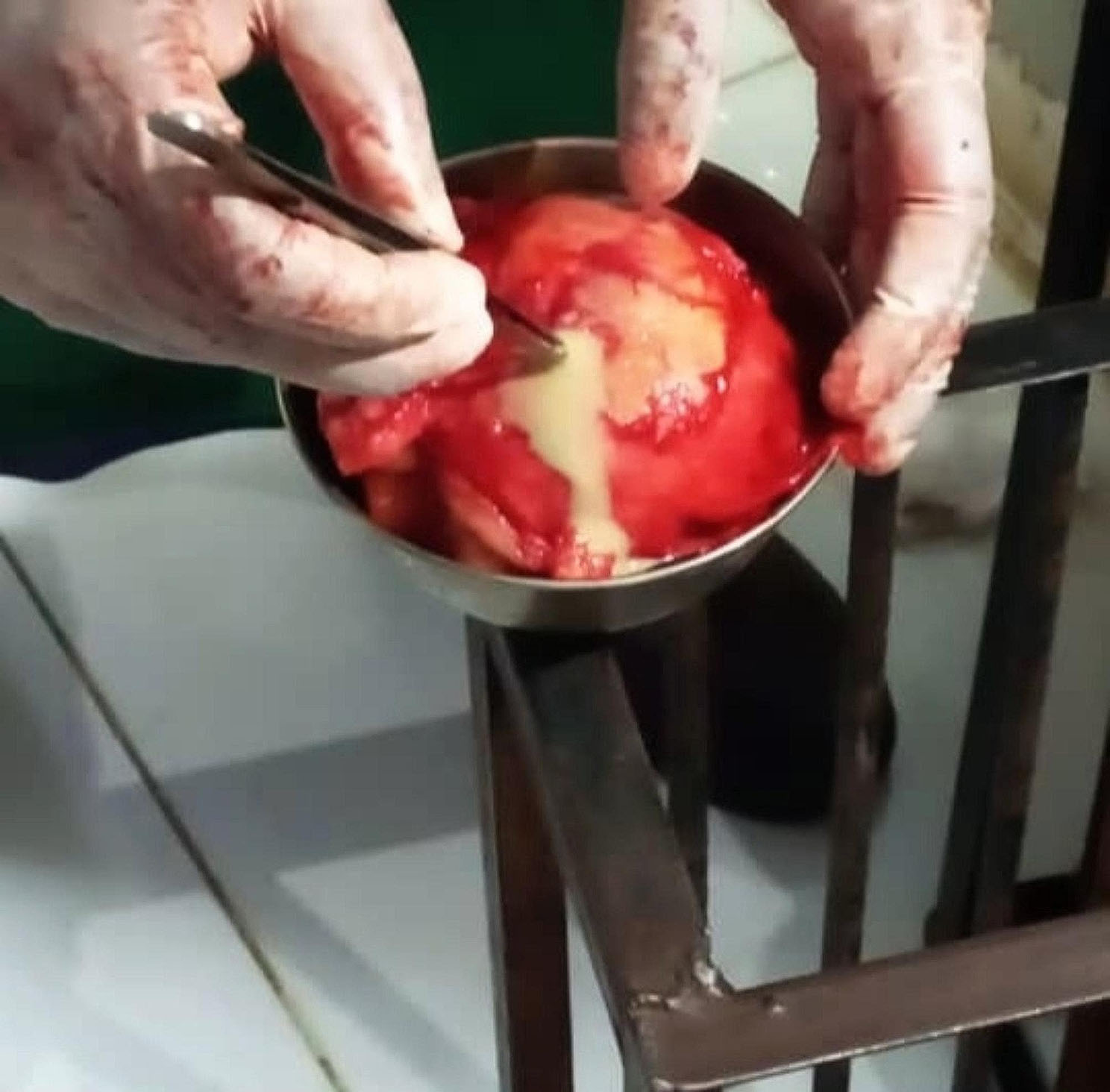
Gossypiboma after taken out

A 37-year-old female, gravida 10 para 10, had a previous emergent caesarean section after abdominal pain for one week. There was no fever, burning urination, or chest pain, and there was no history of ovarian cysts. On examination, she looked vitally stable; the abdomen was distended with mild generalized tenderness, and there was no guarding or rigidity; deep palpation revealed globular mass. Her total white blood cell count was 7.5 cell/µL, hemoglobin was 14 g/dL, random blood glucose was 100 mg/dL, and abdominal ultrasound revealed a mass (10 × 10 cm²) that had been diagnosed as an ovarian cyst. The patient was optimized before being taken to the theatre. Under general anaesthesia, a midline incision was made with a scalpel and unipolar diathermy, subcutaneous tissue was cut, and the muscle was separated. A pink mass was identified; it was adherent to the ovary and jejunum with serosal attachment; it was firm in consistency and measured 10 × 10 cm². The adhesions were released by scissors. The mass was removed and the serosal layer was closed by an interrupted suture. Then the abdominal cavity was washed, after which the drain was placed and the wound was closed in layers. The macroscopic examination revealed a cyst filled with gauze, surrounded by mixed inflammatory cellular infiltration (Figs. 1 and 2). The histopathologist reported no malignant features. In the postoperative course, the patient received intravenous fluid and antibiotics for five days, and she recovered uneventfully from the surgery; she was discharged from the hospital five days after the surgery.

## Discussion

To our knowledge, this is the first reported case of gossypiboma in Sudan in 2024.Despite many previous studies discussing gossypiboma as one of the medical errors occurring in surgery, there is still evidence of the prevalence of such errors worldwide. In Sudan, this issue may be addressed by conducting a root cause analysis to investigate all causes of this phenomenon and addressing them accordingly.

Gossypibomas is a major concern especially in the African countries where incidents of such cases are keep appearing year after year with serous and life threatening complications in some of them [9]. Here, we analyse various aspects of our reported case by comparing it to similar cases reported in Africa over the past five years, in order to offer a broader contextual understanding of the situation. The presented case involves a 37-year-old multiparous female with a history of ten pregnancies and ten previous deliveries, presenting with abdominal pain and a history of an emergent caesarean section. Clinical examination, laboratory findings, and imaging studies led to the diagnosis of an ovarian cyst. However, subsequent surgical exploration revealed an unexpected and intriguing finding - a cyst filled with gauze adherent to the ovary and jejunum. The initial clinical presentation of the patient with abdominal pain and a palpable mass raised suspicion for an ovarian cyst, a common gynaecological condition. However, the unusual discovery during surgery emphasizes the importance of careful preoperative evaluation and thorough exploration in the operating room. The nature of the mass, filled with gauze, raises questions about its origin and how it became adherent to both the ovary and jejunum. A detailed history may provide clues, such as any previous surgical interventions, gynaecological procedures, or abdominal surgeries. The presence of gauze suggests the possibility of a retained surgical sponge, a rare but well-documented occurrence in the literature. The macroscopic examination, as illustrated in Figs. 1 and 2 shows the cystic structure surrounded by mixed inflammatory cellular infiltration. The absence of malignant features reported by the histopathologist is reassuring, ruling out neoplastic aetiologies. A similar case was reported in Sudan in 2023, involving a 24-year-old female with a history of emergency caesarean section. which was also presented after two years of the operation however it was complicated by the development of abdominal obstruction. the difference in age and presentation requires more care from the practitioner when confronted with a suspectable case of gossypiboma [10]. while laparoscopic surgeries are usually considered safer alternatives when compared with classical surgery, there has about been report before about missed gossypibomas after laparoscopic abdominal surgeries, which means that care should also be taken with this type of surgeries [11].

Another African case of gossypiboma was reported in 2022, where a 31-year-old woman presented to the emergency room with similar complaints after three weeks of a cesarean section. Despite the age difference between these cases, the overall clinical prognosis is the same, as most patients show improvement after the foreign body is removed [12]. The management of such cases involves a multidisciplinary approach. The surgical team, in collaboration with gynecologists and general surgeons, must navigate the intricacies of the anatomy involved and carefully dissect adhesions to achieve complete excision. The adherence of the mass to the jejunum introduces an additional layer of complexity, requiring meticulous surgical skills to avoid inadvertent injuries. Postoperatively, the patient’s recovery was uneventful, supported by intravenous fluids and antibiotics for five days. This successful outcome underscores the significance of prompt recognition and intervention in unexpected surgical findings. Contrary to our situation, the prolonged presence of gossypiboma can put pressure on adjacent organs, potentially causing them to perforate. This was highlighted in a recent study by Berhanu N. Alemu and Abraham G. Tiruneh, who documented a case of a patient experiencing abdominal discomfort one year after a cesarean section. Further investigation revealed that the discomfort was due to a gossypiboma leading to a perforated cecum. Timely recognition and intervention could significantly reduce the risk of such complications occurring [7]. This case and similar African-reported cases of gossypiboma underscore the diagnostic challenges that can arise in routine clinical practice. The unexpected discovery of a cyst filled with gauze during surgery emphasizes the need for thorough exploration and a high index of suspicion for retained surgical items.

Preventing retained lap sponges and gossypibomas in the operating theatre requires a multifaceted approach, emphasizing stringent protocols, personal accountability and a collaborative environment [13]. One critical measure is the meticulous instrument and sponge counts conducted at multiple stages: before the procedure, during the procedure, and before wound closure. A standardized checklist, as part of the surgical safety protocol, ensures that all items are accounted for. The atmosphere in the operating room must support a culture of safety where any team member, regardless of hierarchy, feels empowered to challenge the surgeon or operating staff if discrepancies are noted [14]. For instance, if a sponge count is incorrect or an instrument is unaccounted for, member of the operating team should confidently raise the issue without fear of reprimand. Moreover, it was found that these safety practices has to be adjusted according to the nature of the operation and the number of the available staff to ensure the prevention of these events [15].

Advanced technologies such as radiofrequency identification (RFID) or bar-coded sponges can further enhance detection and accountability. Real-world examples of prevention include implementing mandatory “time-out” procedures where the team collectively reviews the count and confirming the absence of retained materials through intraoperative imaging when necessary. This integrated approach significantly reduces the risk of retained surgical items and promotes a culture of shared responsibility for patient safety.

Multidisciplinary collaboration and careful surgical management are essential for optimal patient outcomes in such unusual cases. Future considerations may include a review of protocols to prevent retained surgical items and enhanced communication among healthcare teams to minimize such occurrences.

## Conclusion

To the best of our knowledge, this is the first reported case of gossypiboma in Sudan in 2024. Diagnosed two years post-caesarean section and initially mimicking an ovarian cyst, this case highlights the critical need for rigorous surgical protocols and multidisciplinary approach.

Furthermore, addressing this issue necessitates comprehensive root cause analysis, alongside revisions to healthcare practitioner training and hospital protocols to prioritize error prevention.

The successful management and uneventful recovery of the patient underscore the importance of meticulous preoperative evaluation, thorough intraoperative exploration, and postoperative care. Preventing such occurrences requires stringent instrument and sponge counts, standardized checklists, and a culture where all team members can challenge discrepancies. Adoption of advanced technologies and mandatory “time-out” procedures further enhances prevention efforts. This case underscores the necessity for continuous review and improvement of surgical safety protocols to minimize the risk of retained surgical items and ensure patient safety.

## Data Availability

No datasets were generated or analysed during the current study.
